# Outcrossing rates in an experimentally admixed population of self-compatible and self-incompatible *Arabidopsis lyrata*

**DOI:** 10.1038/s41437-021-00489-8

**Published:** 2021-12-16

**Authors:** Christina Steinecke, Courtney E. Gorman, Marc Stift, Marcel E. Dorken

**Affiliations:** 1 Department of Biology, Trent University, 1600 West Bank Drive, K9J 0G2 Peterborough, ON Canada; 2 Department of Biology, University of Konstanz, Universitätsstrasse 10, 78457 Konstanz, Germany; 3 Present Address: Department of Biology, Queen’s University, 116 Barrie Street, K7L 3N6 Kingston, ON Canada

**Keywords:** Plant evolution, Self incompatability, Ecological genetics, Population genetics

## Abstract

The transition to self-compatibility from self-incompatibility is often associated with high rates of self-fertilization, which can restrict gene flow among populations and cause reproductive isolation of self-compatible (SC) lineages. Secondary contact between SC and self-incompatible (SI) lineages might re-establish gene flow if SC lineages remain capable of outcrossing. By contrast, intrinsic features of SC plants that reinforce high rates of self-fertilization could maintain evolutionary divergence between lineages. *Arabidopsis lyrata* subsp. *lyrata* is characterized by multiple origins of self-compatibility and high rates of self-fertilization in SC-dominated populations. It is unclear whether these high rates of selfing by SC plants have intrinsic or extrinsic causes. We estimated outcrossing rates and examined patterns of pollinator movement for 38 SC and 40 SI maternal parents sampled from an admixed array of 1509 plants sourced from six SC and six SI populations grown under uniform density. Although plants from SI populations had higher outcrossing rates (mean *t*_m_ = 0.78 ± 0.05 SE) than plants from SC populations (mean *t*_m_ = 0.56 ± 0.06 SE), outcrossing rates among SC plants were substantially higher than previous estimates from natural populations. Patterns of pollinator movement appeared to contribute to lower outcrossing rates for SC plants; we estimated that 40% of floral visits were geitonogamous (between flowers of the same plant). The relatively high rates of outcrossing for SC plants under standardized conditions indicate that selfing rates in natural SC populations of *A. lyrata* are facultative and driven by extrinsic features of *A. lyrata*, including patterns of pollinator movement.

## Introduction

The shift from self-incompatibility to self-compatibility is one of the most frequent evolutionary transitions in flowering plants (Barrett 2002; Wright et al. 2013). This transition is associated with high rates of speciation (Goldberg et al. 2010), at least in part because selfing within populations of self-compatible (SC) plants is associated with assortative mating and reduced gene flow among populations (Hamrick and Godt 1996; Martin and Willis 2007; Fishman et al. 2014). Therefore, selfing can lead to reproductive isolation between SC and self-incompatible (SI) lineages (Charlesworth and Pannell 2001). For example, obligately outcrossing and predominantly (but not obligately) selfing populations of *Clarkia xantiana* rarely hybridize in zones of sympatry, resulting in nearly complete reproductive isolation (Briscoe Runquist et al. 2014). However, the extent to which restricted gene flow is driven by the ability to self-fertilize per se or phenotypic divergence that arises after the origin of self-compatibility has rarely been examined.

The initial divergence of SI and SC lineages might be enabled by strong assortative mating within SC lineages for which high rates of selfing could yield reproductive isolation (Cutter 2019). However, if selfing is facultative and its maintenance driven by transient advantages to SC lineages, the transition to self-compatibility might not directly contribute to reproductive isolation. For example, strong genetic differentiation between SC and SI populations of *Linaria cavanillesii* appears to have been driven by historical events, including population bottlenecks, rather than by restricted gene flow arising from high rates of selfing (Voillemot and Pannell 2017). In some groups, the breakdown of self-incompatibility has been followed by phenotypic changes to flowers and inflorescences that reinforce assortative mating within newly-arisen SC lineages. These phenotypic features include reduced flower size, reduced investment in pollinator attraction, and reduced spatial separation between male and female sex organs within flowers, that together represent components of the “selfing syndrome” (Sicard and Lenhard 2011) and that promote reproductive isolation between SC and SI plants (Cutter 2019). Understanding whether reproductive isolation is caused by assortative mating following the transition to SC, or by ancillary features (e.g., traits associated with the selfing syndrome) will clarify the mechanisms underlying high rates of diversification following transitions from SI to SC.

We examined the potential contribution of self-compatibility to reproductive isolation by examining patterns of mating among SI and SC lineages of *Arabidopsis lyrata* subsp. *lyrata* (L.) O’Kane and Al-Shehbaz grown under experimental admixture. This plant is a short-lived perennial native to the eastern Great Lakes region of North America (Mable et al. 2005). Most populations consist of obligately outcrossing SI individuals (Mable et al. 2005). However, in at least six populations, all individuals are SC with low outcrossing rates (*t*) ranging from 0.09 to 0.41 (mean ± standard deviation *t* = 0.24 ± 0.14; Mable et al. 2005; Foxe et al. 2010). These selfing populations likely have at least two recent (<10,000 years ago), independent evolutionary origins. Aside from reduced pollen:ovule ratios, the flowers of plants in SC populations do not display the features typical of the selfing syndrome (Carleial et al. 2017). However, it is still unclear whether low rates of outcrossing in SC populations of *A. lyrata* are driven by as yet unknown (intrinsic) features of SC phenotypes that result in high rates of autonomous selfing, or whether selfing rates are determined by extrinsic properties of SC populations. Such extrinsic properties could include aspects of plant-pollinator interactions that affect the movement of pollen between flowers of the same plant (i.e., geitonogamous pollen movement) versus between plants (i.e., xenogamous pollen movement). They could also include demographic features of SC populations such as low plant densities that might reduce rates of inter-plant movement by pollinators, which is linked to higher selfing rates in some animal-pollinated plants (Chen 2000; Bosch and Waser 2001; Naito et al. 2008; see also Moeller et al. 2012; Van Etten et al. 2015). If intrinsic plant features drive rates of selfing in *A. lyrata*, one would expect high selfing rates to be maintained when SC and SI populations are brought into secondary contact.

Here, we aimed to determine whether, under standardized conditions, SC plants maintain lower outcrossing rates than SI plants, and if so, whether this might construe a reproductive barrier between SC and SI populations. To do this, we used plants originally sourced from six SI and six SC populations of *A. lyrata* and grew them at high densities under experimental admixture. To determine the contribution of geitonogamous pollination to rates of selfing for SC plants, we also examined patterns of intra- versus inter-plant pollinator movement. This study represents one component of a series of tests of mechanisms that might drive reproductive isolation among SC and SI lineages of *A. lyrata* (Gorman et al. 2020a, 2021b). Those companion studies showed that: (1) flowering times broadly overlap for plants from SC and SI populations (Gorman et al. 2020a); (2) pollinators do not discriminate between plants from SC and SI populations and tend to move to nearest-neighbor inflorescences when foraging (Gorman et al. 2020a); and (3) there are no post-pollination reproductive barriers between plants from SC and SI populations (Gorman et al. 2021b). Together with those other findings, high outcrossing rates for SC plants under experimental admixture would imply that secondary contact between SC and SI lineages would prevent reproductive isolation between lineages. High rates of outcrossing for SC plants would also indicate that extrinsic features cause the high selfing rates observed in SC populations of *A. lyrata*. By contrast, low rates of outcrossing for SC plants under experimental admixture would indicate that intrinsic features associated with self-compatibility might reinforce assortative mating in SC populations. If so, the findings (1–3) listed above may not preclude reproductive isolation between SC and SI lineages of *A. lyrata* and further evolutionary divergence.

## Materials and methods

### Study species

*Arabidopsis lyrata* subsp. *lyrata* (Brassicaceae) is a small, insect-pollinated, short-lived perennial native to the Great Lakes region of North America. It grows in relatively dry habitats with porous soils, such as sand dunes and rocky outcrops (Mable et al. 2005). Like many other Brassicaceae, this plant is usually characterized by sporophytic SI (Mable et al. 2003) and thus obligately outcrossing, although hand-pollinations have indicated that SC individuals occur at low frequencies in otherwise SI populations (Mable et al. 2005). A few populations consist of only SC plants and are characterized by a mating system with high selfing rates (Foxe et al. 2010) and shorter-life spans (Gorman et al. 2020b). Evidence suggests that there have been at least two relatively recent (<10,000 years ago) independent transitions to selfing (Hoebe et al. 2009; Foxe et al. 2010; Mable et al. 2017). In line with this recent origin, the SC populations have not evolved a clear selfing syndrome (Carleial et al. 2017). There is no evidence that pollinators discriminate between mating types (Gorman et al. 2020a).

Flowers on *A. lyrata* inflorescences have overlapping periods of stigma receptivity and pollen dehiscence, but SC plants do not produce seeds in the absence of pollinators (e.g., when grown in growth chambers). Although this means that selfing is not autonomous for SC plants, the close physical proximity of the anthers and stigma (Carleial et al 2017) can easily result in autogamous self-pollination (e.g., during a pollinator visit). Indeed, when conducting hand crosses, bud-emasculations of recipient SC plants are needed to prevent contamination by self-pollen (Li et al. 2019). In addition to (facilitated) autogamous self-pollination, pollinators can cause geitonogamous selfing when moving among flowers within *A. lyrata* plants, which often display multiple flowers (in this study, plants displayed between 1 and 46 flowers at a time) and pollinators frequently move among flowers within plants (Gorman et al. 2020a).

### Common garden experiment

Our study is based on a common garden experiment (described in more detail in Gorman et al. 2020a, b) designed to test a series of hypotheses related to the occurrence of reproductive isolation between SC and SI lineages of *A. lyrata*. The experimental plants were the product of crosses made between field-collected seeds from North American *A. lyrata* populations with known breeding and mating systems. These populations represented six SC populations (Foxe et al 2010), and six SI populations selected for their close geographic proximity or comparable latitude to the SC populations. The whole experiment included additional cross-types, but in this study we exclusively used plants from SC cross-types (SC × SC_within_ and SC × SC_between_, i.e., progeny of within- and between-population crosses among plants from the five SC populations) and SI cross-types (SI × SI_within_ and SI × SI_between_, i.e., progeny of within- and between-population crosses among plants from the five SI populations). Testing our main hypothesis required comparisons between breeding systems, not between different cross-types within each breeding system. Nevertheless, as explained in more detail below, we initially accounted for within- versus between-population cross-types in our statistical models. Additional information on these crosses is provided in the Supplementary Materials (Supplementary Tables S1 and S2).

Plants for the experiment were propagated by first sowing seeds from each of the within- and between-population cross-types listed above in peat-based substrate in individual 3.5-inch pots between March 20 and 22, 2018. Seeds were germinated in microclimate chambers (Conviron TCR, Manitoba, Canada) until seedlings produced two true leaves. Microclimate conditions followed 11-h days with a 21/18 °C day/night cycle at 95% humidity. Up to three haphazardly chosen seedlings from each seed family were transplanted into individual Ray Leach “Cone-Tainers”™ (Tangent, Oregon, USA) between April 18 and May 1, 2018. The plants were moved to the common outdoor garden located at Trent University (Peterborough Ontario) on May 10 (prior to flowering) and placed into positions following a randomized block-design (Gorman et al. 2020a). A total of 1509 individuals were located in three replicate 1 × 9 m blocks (1, 2, and 3), and each block was subdivided into three quadrats (A, B, and C), yielding a total of nine quadrats (1A, 1B, 1C; 2A, 2B, 2C; and 3A, 3B, 3C). Each block contained 9 trays of cone-tainers, each with approximately 20 cone-tainers, yielding up to 180 equidistantly spaced plants per 3 m^2^ quadrat (60 plants/m^2^). Each seed family and cross-type was randomly distributed within and between quadrats (Gorman et al. 2020a).

The main flowering period was between June 1 and July 14, 2018. The date of peak flowering was similar among cross-types, which also had broadly overlapping flowering phenologies (Gorman et al. 2020a). On the date of peak flowering, plants produced an average of 12 flowers per day, and there were no strong differences in the maximum number of flowers produced among cross-types (Gorman et al. 2020a). The predominant floral visitors were solitary bees and hoverflies, and both types of pollinators visited SC and SI plants at similar frequencies (Gorman et al. 2020a).

### Seed collection

To estimate outcrossing rates for plants from SC and SI cross-types, we sampled 78 plants (38 SC plants from 21 SC × SC_within_ and 17 SC × SC_between_, and 40 SI plants from 14 SI × SI_within_ and 26 SI × SI_between_) from the common garden experiment. Seed families were sampled by collecting one ripe fruit from a randomized subset of plants from the first block of the experimental garden (block 1). Because mature fruits representing the cross-types in our sampling scheme were not always available during the period over which seeds were collected (July 10–31, 2018), cross-types were unevenly represented in our final sample. Collected fruits were placed individually in 1.5 ml microtubes, which were left open to dry at room temperature for 5–7 days before they were sealed and stored in a refrigerator at 4 °C until use.

### DNA extraction and genotyping

Beginning on August 21, 2018, seeds were removed from fruits and soaked in distilled water for up to 24 h. The seed coat was then removed from each seed prior to being ground into a semi-fine powder using micro pestles. DNA was extracted using QuickExtract™ Plant DNA Extraction Solution (Lucigen, Wisconsin, USA) following the manufacturer’s instructions, and eluted to a final volume of 50 µl. DNA was then purified using E.Z.N.A plant DNA kits (Omega Bio-tek, Georgia, USA) following the manufacturer’s instructions, which were modified so that we began with the filtration and DNA precipitation step. Final eluted volumes were 60 µl.

To estimate outcrossing rates, we genotyped seeds at four simple sequence repeat loci (athzfpg, atts0392, lyr417, adh-1; Clauss et al. 2002; Mable and Adam 2007) previously used for mating-system estimation for *A. lyrata* (Foxe et al. 2010). The forward primers were fluorescently labeled with either hex (atts0392, lyr417, adh-1) or 6-fam (athzfpg). DNA amplifications were conducted as single reactions with a final volume of 10 µl. Three of the four amplification reactions (atts0392, lyr417, adh-1) involved the use of 3.55 µl dH_2_O, 2 µl 5 × colorless GoTaq™ reaction buffer (Promega, Madison Wisconsin), 1 µl BSA, 0.8 µl MgCl_2_, 0.2 µl dNTPs, 0.1 µl forward primer, 0.1 µl reverse primer, 0.25 µl GoTaq™ DNA polymerase (Promega), and 2 µl DNA template. Amplification for these three loci involved the following run conditions: denaturation at 95 °C for 2 min, followed by 35 cycles of denaturation at 95 °C for 45 s, annealing at 57 °C for 30 s, extension at 72 °C for 1 min, and a final extension of 72 °C for 5 min. The fourth reaction (athzfpg) involved the use of 2.1 µl dH_2_O, 2 µl 5 × colorless GoTaq™ reaction buffer (Promega), 2 µl BSA, 1.2 µl MgCl_2_, 0.2 µl dNTPs, 0.1 µl forward primer, 0.1 µl reverse primer, 0.3 µl GoTaq™ DNA polymerase (Promega), and 2 µl DNA template. This fourth locus was amplified using the following run conditions: denaturation at 95 °C for 2 min, followed by 40 cycles of denaturation at 95 °C for 45 s, annealing at 55.8 °C for 30 s, extension at 72 °C for 1 min, and a final extension of 72 °C for 5 min. All amplification reactions were conducted using MasterCycler epGradient thermocyclers (Eppendorf, Hamburg, Germany).

Amplification products were diluted (1:10 dilutions) and genotyped using an automated sequencer (Applied Biosystems 3730 DNA analyzer, Applied Biosystems, Foster City, California) with ROX500 (Applied Biosystems) size standard for reference. Genotypes were analyzed using GeneMapper*ID-X* software (v. 4.0 Applied Biosystems). Genotype scores were corrected manually after visual inspection of electropherograms. In some cases, amplification failed or provided equivocal allele scores. These seeds were excluded from analysis, yielding a total sample of 800 seeds from 77 seed families (38 SC and 39 SI).

### Outcrossing rate estimation

Rates of outcrossing (*t*) were estimated using the MLTR software program (v. 3.4; Ritland 2002). This program uses a maximum likelihood procedure to estimate *t* from estimated allele frequencies in the population and inferred maternal genotypes (i.e., inferred from the segregation of alleles among progeny from the same seed parent). The calculations assume mixed-mating, with selfing occurring at a rate *s*, where *s* = 1 – *t*_m_, and where the subscript *m* refers to estimation of *t* across multiple loci. Our estimates of *t*_m_ were made at the individual level using 10–12 seeds per plant for a total sample of 800 seeds from 77 seed families (38 SC and 39 SI). As a low frequency of null alleles had been reported for our loci (Foxe et al. 2010), we ran the MLTR program assuming that null alleles were present.

### Patterns of insect visitation

Opportunities for self-pollination via the transfer of pollen among flowers within plants (i.e., via geitonogamous pollen transfer) were investigated by recording videos of the movement patterns of floral visitors (hereafter referred to as pollinators) in small arrays of plants. In each array, four to six plants (depending on their size) were taken from the randomized blocks that formed part of the larger common garden experiment (so this sample of plants therefore included plants not sampled for outcrossing rate estimation), such that the plants that formed an array were chosen randomly regardless of the cross-type and populations they represented (for more details on these methods, see Gorman et al. 2020a). Before starting each video segment, the number of simultaneously open flowers per plant in the array was recorded. To avoid potential effects of disturbance associated with starting and stopping the recordings, videos were trimmed to a 10-min segment that excluded the beginning and end of the recording. A total of 140 videos were analyzed, representing 23.3 h of video footage from 379 plants, 123 of which were included in more than one video recording. For each video, we recorded the type of pollinator (distinguishing between the categories solitary bee and hoverfly), and the associated duration of the visits. In addition, for each visitor, we recorded the path taken through the array from the first flower they visited; from that flower we tracked whether pollinators moved to another flower on the same plant, or to a flower on a different plant. We tracked this pattern of within- versus between-plant pollinator movements until the pollinator left the video frame. On average, each flower received 2.07 ( ± 0.13 SE) visits per hour and pollinators displayed no preference for SC versus SI cross-types (Gorman et al. 2020a). Movements outside of the video frame were tallied as between-plant movements. Based on these movement patterns, we calculated the proportion of within- versus between-plant pollinator movements for each plant and used the average values as empirical estimates of *p*_g_ and *p*_x_ for the calculations described below.

### Floral display and opportunities for geitonogamy

We used our observations of pollinator movements within versus between inflorescences to estimate the total proportion of geitonogamously visited flowers per plant. To do this, we assumed that geitonogamous pollination depended on the frequency of within- versus between-plant pollinator movements, that each pollinator movement was independent of inflorescence size, flower position, and of previous movements (i.e., pollinators moved xenogamously to another plant with fixed probability *p*_x_, or visited another flower on the same plant with probability *p*_g_ = 1 – *p*_x_), that each flower had an equal probability of receiving an initial pollinator visit, that pollinators did not re-visit flowers on an inflorescence, and therefore that the number of pollinator visits per plant per day was finite and limited by the total number of open flowers per day. In practice, probabilities *p*_g_ and *p*_x_ are likely to vary among plants that, for example, were visited by different types of pollinators (Brunet and Sweet 2006) or had different numbers of flowers (Harder and Barrett 1995); however, our observations were not sufficiently detailed to include these aspects of geitonogamous pollination. Based on the observations described in more detail in Gorman et al. (2020a), we further assumed that pollinators did not preferentially visit particular cross-types or otherwise alter their behavior when visiting different cross-types. Using these assumptions, the empirical estimates of *p*_g_ and *p*_x_ based on patterns of insect visitation, and the daily record of open flowers per plant (Gorman et al. 2021a), we calculated the daily per-plant probability of geitonogamy *π* as follows:

$$\pi = p_g \cdot p_x + p_g^2 \cdot p_x + p_g^3 \cdot p_x + \cdots + \,p_g^{n - 1} = 1 - p_x,$$

where *n* is the number of open flowers per plant per day (also see Supplementary Methods). Daily values of *π* were therefore equal to 0 (for plants with fewer than two open flowers) or *p*_g_ (for plants with more than two flowers). We used these daily estimates of *π* to calculate the average proportion of geitonogamously visited flowers per plant, *G* as follows:

$$G = \frac{{\mathop {\sum}\nolimits_{i = 1}^d {f_i\pi _i} }}{{\mathop {\sum}\nolimits_{i = 1}^d {f_i} }},$$

where *d* is the total number of days over which each plant produced multiple flowers and *f* the number of open flowers per plant on day *i*, such that *G* is the weighted average of *π*. Put another way, values for *G* for a given plant represent the average per-visit probability of geitonogamy (*π*) scaled to reflect variation in the number of flowers displayed per day. Under this approach values of *G* increased with average display size and reached a maximum of *G* = *p*_g_ for plants that always displayed multiple flowers.

### Statistical analyses

To evaluate differences in outcrossing rates between plants from SC and SI populations, we first used linear mixed-effects model with *t*_*m*_ as the dependent variable, cross-type (SI × SI_within_, SI × SI_between_, SC × SC_within_, SC × SC_between_) as the independent variable, and maternal ID (seed family) as a random grouping variable. We used a contrast-matrix approach to evaluate the overall difference in outcrossing rates between plants from SC versus SI populations using the “contrasts” option in the lmer function. This analysis indicated that there were no differences in outcrossing rates between cross-types representing the same breeding system (i.e., no differences between SI × SI_within_ and SI × SI_between_, or between SC × SC_within_ and SC × SC_between_; Supplementary Materials). Accordingly, and because we had no a priori reason to expect differences in outcrossing rates between cross-types with the same breeding system, we simplified this analysis so that breeding system (SI versus SC) was the independent variable. Mixed-model parameters were calculated using the lmer function from the lme4 package (v. 1.1–27; Bates et al. 2015) in R (v. 4.1.0; R Core Team 2021) and significance of fixed effects (the SC versus SI contrast) was assessed using type-2 tests obtained from the Anova function from the car package (v. 3.0–10; Fox and Weisberg 2019).

We used permutation tests to evaluate the extent to which any observed differences in outcrossing rates between plants from SC and SI populations may have been driven by the particular sample of seed families in our subsample of plants from the larger experiment. In each of 5000 permutations, we randomly sampled (with replacement) outcrossing rates from 800 seeds (400 from each of the SC and SI cross-types). While resampling data points, all other information related to the sampled data was retained, including the breeding system of the maternal parent and the maternal parent’s source population. We used these resampled datasets to re-run the mixed-model calculations using the same procedure as described in the previous paragraph. From these bootstrapped analyses, we report the upper and lower 95% confidence intervals for the difference in mean *t*_m_ between SC and SI and test-statistic values of mixed-model analyses of permuted data, and present the distribution of *P* values in the Supplementary Materials (Supplementary Fig. S1).

We evaluated whether plants from SI versus SC cross-types differed in terms of their expected probability of geitonogamous pollination using a linear mixed-effects model with *G* as the dependent variable, breeding system (SI versus SC) as the independent variable, and maternal ID (seed family) as a random effect using the lmer function. Significance of the fixed effect was evaluated using the Anova function, as in the analysis of outcrossing rates outlined above.

## Results

**Fig. 1:**
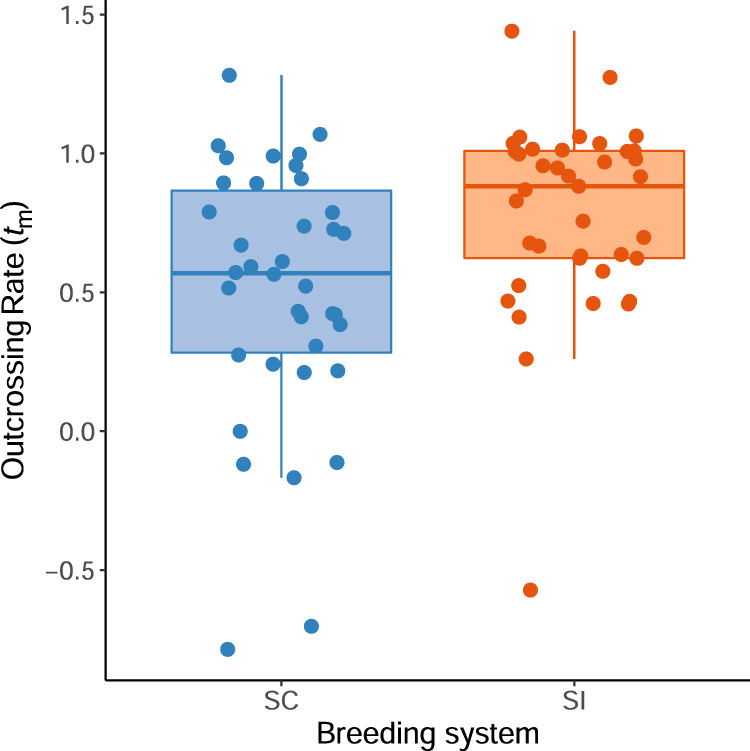
Variation in family-level estimates (circles) of outcrossing rates (*t*_m_) among self-compatible (SC) and self-incompatible (SI) cross-types of *Arabidopsis lyrata* grown in a common garden. Box plots indicate the median *t*_m_ for each cross-type (horizontal black line in each box), the first and third quartiles (the lower and upper edge of each box), and the minimum and maximum values of *t*_m_ that are no further than 1.5 the inter-quartile range (the whiskers extending from each box).

SC plants had outcrossing rates of *t*_m_ = 0.56 ± 1.12 SD compared to outcrossing rates of *t*_m_ = 0.78 ± 0.99 SD for SI plants (see Fig. 1 for family-level mean *t*_m_). This difference in outcrossing rates between breeding systems was statistically significant (linear mixed-model parameter estimate for SC versus SI plants: 0.24 ± 0.09 SE; type-2 Wald Chi-square test: *χ*^2^ = 7.31, *P* = 0.007). However, the distribution of permuted differences in mean outcrossing rates between plants from SC versus SI cross-types covered a broad range (95% CI for the difference in *t*_m_ between SC and SI plants: 0.08–0.37), and for more than half of these bootstrapped analyses, the associated *P* values from the mixed-model analyses were not significant (i.e., they were greater than or equal to 0.05; Supplementary Fig. S1). Therefore, our finding of a significant difference in outcrossing rates between SC and SI cross-types appeared to have been driven, at least in part, by the particular sample of seed families collected for analysis.

**Fig. 2:**
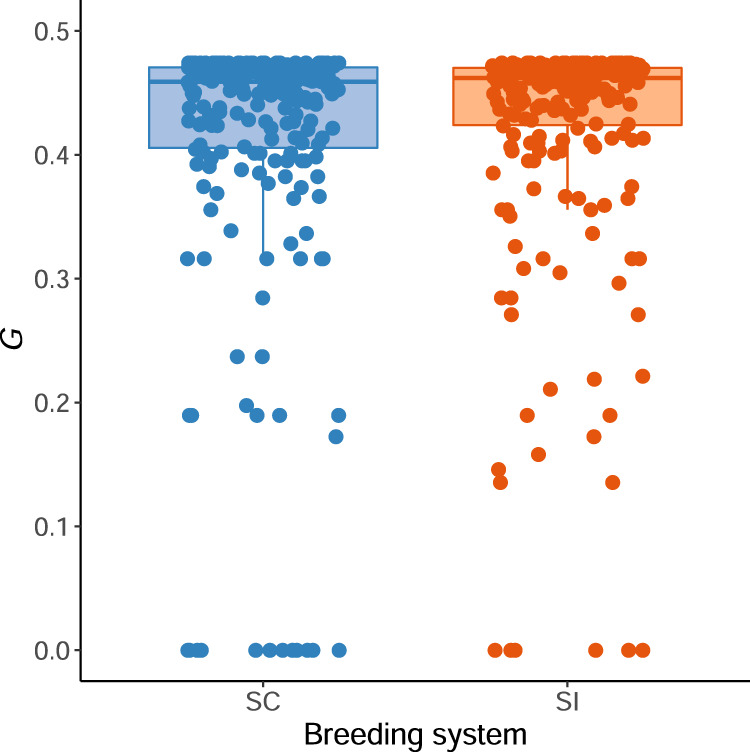
Variation in the average expected probability of geitonogamous pollinator visits (*G*) for self-compatible (SC) and self-incompatible (SI) plants in a common garden of *Arabidopsis lyrata*. Note that the apparent upper bound arises because *G* could not exceed 1 – *p*_x_, where *p*_x_ is the per-visit probability that pollinators leave an inflorescence after visiting the first flower (see Methods for further details).

The proportion of intra-plant pollinator movements (*p*_g_) was 0.47, indicating that, on average, approximately half of all initial pollinators visits were followed by visits to another flower on the same inflorescence. Values of *G* were similar in magnitude between breeding systems (mean *G* ± SE for SI plants: 0.41 ± 0.01; SC plants: 0.40 ± 0.01; Fig. 2; linear mixed-model parameter estimate: 0.02 ± 0.01 SE; type-2 Wald Chi-square test: *χ*^2^ = 1.51, *P* = 0.22). Thus, on average, we expect that plants from both breeding systems were subjected to similar patterns of within- versus between-plant pollinator movement.

## Discussion

Not surprisingly, average values of outcrossing tended to be lower among SC compared to SI cross-types. However, there was substantial variation in rates of outcrossing among SC plants and average outcrossing rates for SC plants exceeded those found in natural populations of SC *A. lyrata* (cf. our value of *t*_m_ = 0.56 with an average *t*_m_ = 0.27 ± 0.05 SE among eight predominantly SC populations in Foxe et al. 2010; see also Mable et al. 2005; Mable and Adam 2007; Willi and Määttänen 2010). By contrast, average outcrossing rates for SI plants in our experiment fell within the range of those reported from entirely SI populations (Foxe et al. 2010). Higher rates of outcrossing among SC plants in our experimental garden where plants were grown at high densities and with abundant pollinators indicate that the lower rates of outcrossing detected in natural populations are not driven by intrinsic features of SC plants, but rather by extrinsic features related to ecological processes operating in those populations. Moreover, the magnitude of self-fertilization among plants from SC populations in our experiment closely corresponded with the estimated frequency of within- versus between-plant pollinator movements, indicating that selfing rates in SC *A. lyrata* might be driven in large part by patterns of pollinator behavior. Substantial rates of outcrossing for SC and SI cross-types, combined with an absence of assortative pollinator movements within cross-types (Gorman et al. 2020a), argue against the possibility that the capacity to self-fertilize is a barrier to gene flow between SI and SC lineages of *A. lyrata*. Below, we discuss these results, and their implications for the maintenance of reproductive isolation between SC and SI lineages of *A. lyrata*.

Studies of natural populations of North American *A. lyrata* have repeatedly shown low levels of outcrossing in SC populations (Mable et al. 2005; Mable and Adam 2007; Foxe et al. 2010; Willi and Määttänen 2010). Although outcrossing rates among SC plants in our study were highly variable, they exceeded all previous estimates from natural SC populations. Indeed, our permutation test usually failed to recapitulate the significant difference in mean outcrossing rates between SC and SI cross-types. The capacity for substantial rates of outcrossing found here indicates that the high rates of selfing detected in previous studies of natural populations of *A. lyrata* are not the result of intrinsic features of SC phenotypes. Instead, our results lead to the hypothesis that the high rates of selfing detected in SC populations are the result of extrinsic features of those populations (e.g., plant density, flowering phenology, pollinator abundances, and patterns of pollinator visitation). In terms of plant densities, our experimental population was maintained at a higher density than found in nature (60 plants/m^2^ in the common garden versus ~7.2 plants/m^2^ on average in natural populations; Mable and Adam 2007, and see Willi et al. 2013). In general, plants that occur in dense patches often receive higher rates of pollinator visitation (Klinkhamer and de Jong 1990; Grindeland et al. 2005; Hegland and Totland 2005; Bernhart et al. 2008) and this can affect rates of selfing. For example, SC populations of *Clarkia xantiana* occur at lower plant densities and have lower rates of pollinator visitation than SI populations of *C. xantiana* across a contact zone that separates the two mating phenotypes (Fausto et al. 2001). Accordingly, selection for features that enable reproductive assurance via selfing (e.g., reduced herkogamy and protandry) was strongest in the populations where autogamous selfing is fostered (Moeller and Geber 2005). Low population densities and pollen limitation may similarly underlie high rates of selfing in natural SC populations of *A. lyrata*.

Patterns of flowering phenology may have further contributed to substantial rates of outcrossing for SC plants in the experimental garden. Peak flowering time, the probability of flowering, the maximum number of flowers open per day, and the overall duration of flowering strongly overlapped among SC and SI plants in the common garden experiment (Gorman et al. 2020a). As a result, opportunities for pollen transfer with other plants (outcrossing) were comparable for SC and SI plants (Gorman et al. 2020a). Floral synchrony and mass blooming can affect rates of outcrossing via effects on mate availability (Cruzan et al. 1994; Delmas et al. 2015) and pollinator activity (Yoshiaki and Kudo 2009). High levels of floral synchrony in the common garden experiment may have similarly contributed to higher outcrossing rates of SC cross-types than those observed in natural populations of SC *A. lyrata*.

Patterns of foraging by pollinators, together with the close proximity of mating partners in our garden might have further contributed to the substantial outcrossing rates we detected for SC plants. Pollinators did not show a preference for mating phenotype (SC versus SI) and visitation by both solitary bees and hoverflies was primarily influenced by which plants were nearest neighbors (Gorman et al. 2020a). The apparent absence of pollinator preferences for mating phenotype may have been influenced by the similarity in SC and SI flower morphology (Carleial et al. 2017). However, even among plants for which selfing syndromes are not obvious or have not yet evolved, pollinators can prefer plants from lineages with higher rates of outcrossing. For example, bumblebees were significantly more likely to visit the flowers of outbred lineages of *Mimulus guttatus* (Carr et al. 2015). Moreover, other studies have found that selfing populations are common in areas where pollinators are not frequent (Fausto et al. 2001; Moeller 2006). Geographic patterns of pollinator abundances have not been studied for *A. lyrata*, and so whether similar patterns can help explain rates of selfing in SC populations is unknown.

The self-fertilization that did occur among plants from SC populations in our experimental garden appears to be have been driven by patterns of pollinator behavior. Intra-plant movements accounted for approximately 40% of all between-flower movements by pollinators in the garden and this pattern of foraging should result in geitonogamous pollination (Harder and Barrett 1995; Brunet and Sweet 2006), and therefore higher selfing rates for SC plants (Karron et al. 2009). For SC plants in our experiment, average values of *G* were 0.40. Therefore, our expected rates of geitonogamy can almost fully account for observed rates of selfing by SC plants (cf. *G* = 0.40 with 1 – *t*_m_ = 0.44). That patterns of selfing appear to have been driven by extrinsic features of SC *A. lyrata* populations, including plant densities and patterns of pollinator behavior coincides with the results of a recent study of *Mimulus ringens*. In that study, extrinsic features of populations, including plant densities, the size of floral displays, and patterns of within- versus between-plant foraging by pollinators, not intrinsic features of flowers (e.g., degree of herkogamy), appear to be the main processes driving variation in selfing rates among populations (Christopher et al. 2021). In our experimental garden, the high density of plants together with the occurrence of synchronous flowering (Gorman et al. 2020a) likely contributed to patterns of intra-plant foraging by pollinators, resulting in opportunities for geitonogamy and lower outcrossing rates for plants from SC populations.

Our results, together with those from two companion studies (Gorman et al. 2020a, 2021b), indicate that there are no reproductive barriers between SC and SI lineages of *A. lyrata* upon secondary contact. In particular, as found here, substantial rates of outcrossing among SC plants combined with (1) broad phenological overlap in flowering times for plants from SC and SI populations when grown in a common environment (Gorman et al. 2020a), (2) the observation that floral visitors do not discriminate between SC and SI cross-types and instead tend to move between nearest neighbors (Gorman et al. 2020a), and (3) the absence of post-pollination (or other post-zygotic) reproductive barriers between plants from SC and SI lineages (Gorman et al. 2021b) indicate that the mating patterns observed here would have resulted in substantial gene flow among populations, including between SC and SI lineages. Moreover, as discussed above, our results indicate that self-fertilization in SC *A. lyrata* is facultative and depends on extrinsic features such as patterns of inter-plant pollinator movement. As a result, we conclude that the evolution of self-compatibility—even though it is commonly associated with strong assortative mating in natural populations (high rates of selfing)—does not itself prevent gene flow with SI lineages of *A. lyrata*. For the many lineages of SC plants that have diverged from closely related SI lineages, drivers of reproductive isolation that arose after the evolution of self-compatibility, including traits that reinforce assortative mating (including those associated with the selfing syndrome; e.g., Slotte et al. 2011; Briscoe Runquist et al. 2014), may have been necessary to maintain divergence between SC and SI lineages.

## Supplementary information

Supplemental Material

## Data Availability

The data are available on the Dryad Digital Repository at 10.5061/dryad.bg79cnpc1.
