# Supplementary material for: Outcrossing rates in an experimentally admixed population of self-compatible and self-incompatible Arabidopsis lyrata
Source: Heredity (Edinb). 2021 Dec 16;128(1):56–62. doi: 10.1038/s41437-021-00489-8 (PMC8733029; doi:10.1038/s41437-021-00489-8)
Supplement: Supplementary file 1 — Supplemental Material [file 41437_2021_489_MOESM1_ESM.docx]

**Steinecke C, Gorman CE, Stift M, Dorken ME (2021): Outcrossing rates in an experimentally admixed population of self-compatible and self-incompatible *Arabidopsis lyrata*. Heredity.**

**Supplementary Materials**

**Table of Contents**

Table S1: Crosses made to generate SC plants 2

Table S2: Crosses made to generate SI plants 3

Variation in outcrossing rates among SI and SC cross types 4

Calculation of *π* 5

Distribution of *P*-values from permuted tests of the
difference in outcrossing rates between SC and SI plants 6

Supplementary Table S1. Crosses made among plants from self-compatible populations of *Arabidopsis lyrata*. Population codes follow those from Foxe et al. (2010). Numbers refer specific plants sourced from each of those populations.

|  | FATHER | PTP | | | | | LPT | TSSA | | | | | | | RON | | | | | | | | | | | | | TC | | |
| --- | --- | --- | --- | --- | --- | --- | --- | --- | --- | --- | --- | --- | --- | --- | --- | --- | --- | --- | --- | --- | --- | --- | --- | --- | --- | --- | --- | --- | --- | --- |
| MOTHER |  | 3 | 7 | 8 | 10 | 11 | 3 | 2 | 10 | 16 | 19 | 21 | 23 | 30 | 3 | 4 | 5 | 7 | 10 | 13 | 14 | 17 | 19 | 21 | 27 | 28 | 41 | 19 | 21 | 24 |
| PTP | 7 |  |  |  |  | x | x |  |  |  |  |  |  | x |  |  |  |  |  |  |  | x |  |  |  |  |  |  |  |  |
|  | 8 |  |  |  |  |  |  |  | x |  |  |  |  |  |  |  |  |  |  |  |  |  |  |  |  |  |  |  |  |  |
|  | 10 | x |  |  |  |  |  |  |  |  |  |  |  |  |  |  |  |  |  |  |  |  |  |  |  |  |  |  |  |  |
| LPT | 1 |  |  |  |  |  |  |  |  |  |  |  |  |  |  |  |  |  |  |  |  |  |  |  |  |  |  |  |  |  |
|  | 9 |  |  |  |  |  |  |  |  |  |  |  |  |  | x |  |  |  |  |  |  |  |  |  |  |  |  |  |  |  |
| TSSA | 10 |  |  |  |  |  |  |  |  |  |  | x |  |  |  |  |  |  |  |  |  |  |  |  |  |  |  |  |  |  |
|  | 17 |  |  |  |  |  |  |  |  |  |  |  | x |  |  |  |  |  |  |  |  |  |  |  |  |  |  |  |  |  |
|  | 18 |  |  |  |  |  |  | x |  |  |  |  |  |  |  |  |  |  |  |  |  |  |  |  |  |  |  |  |  |  |
|  | 19 |  |  |  |  |  |  |  |  |  |  |  |  |  |  |  |  |  |  |  |  |  |  |  |  |  |  |  |  |  |
|  | 21 |  |  |  |  |  |  |  | x |  |  |  |  |  |  |  |  |  |  |  |  |  |  |  |  |  |  |  |  |  |
|  | 22 |  |  |  |  |  |  |  |  |  |  |  |  |  |  |  |  |  |  |  |  |  |  | x |  |  |  |  |  |  |
|  | 30 |  | x |  |  |  |  |  |  |  |  |  |  |  |  |  |  |  |  |  |  |  |  |  |  |  |  | x |  |  |
| RON | 2 |  |  |  |  |  |  |  |  |  |  |  |  |  |  |  |  |  |  |  |  |  |  |  |  | x |  |  |  |  |
|  | 3 |  |  |  |  |  |  |  |  |  |  |  |  |  |  |  |  |  |  |  | x |  |  |  |  |  |  |  | x |  |
|  | 4 |  |  |  |  |  |  |  |  |  | x |  |  |  |  |  |  |  |  | x |  |  |  |  |  |  |  |  |  |  |
|  | 5 |  |  |  |  |  |  |  |  |  |  |  |  |  |  |  |  | x |  |  |  |  |  |  |  |  |  |  |  |  |
|  | 7 |  |  |  |  |  |  |  |  |  |  |  |  |  |  |  | x |  |  |  | x |  |  |  |  |  |  |  |  |  |
|  | 10 |  |  |  |  |  |  |  |  |  |  |  |  |  |  |  |  |  |  |  |  |  |  |  | x |  |  |  |  |  |
|  | 14 |  |  | x |  |  |  |  | x |  |  |  |  |  |  |  |  |  |  |  |  |  |  |  |  |  |  |  |  |  |
|  | 16 |  |  |  |  |  |  |  |  |  |  |  |  |  |  |  |  |  |  |  |  |  | x |  |  |  |  |  |  |  |
|  | 17 |  |  |  |  |  |  |  |  |  |  |  |  |  |  |  |  |  |  |  |  |  |  |  |  |  | x |  |  |  |
|  | 21 |  |  |  |  |  |  |  |  |  |  |  |  |  |  | x |  |  |  |  |  |  |  |  |  |  |  |  |  |  |
|  | 27 |  |  |  |  |  |  |  |  |  |  |  |  |  |  |  |  |  | x |  |  |  |  |  |  |  |  |  |  |  |
| TC | 16 |  |  |  |  |  |  |  |  |  |  |  |  |  |  |  |  |  |  |  |  |  |  | x |  |  |  |  |  |  |
|  | 19 |  |  |  |  |  |  |  |  |  |  |  |  | x |  |  |  |  |  |  |  |  |  |  |  |  |  |  |  | x |
|  | 21 |  |  |  |  |  |  |  |  |  |  |  |  |  | x |  |  |  |  |  |  |  |  |  |  |  |  |  |  |  |
|  | 24 |  |  |  |  |  |  |  |  |  |  |  |  |  |  |  |  |  |  |  |  |  |  |  |  |  |  | x | x |  |
|  | 25 |  |  |  |  |  |  |  |  |  |  |  |  |  |  |  |  |  |  |  |  |  |  |  |  |  |  |  | x |  |
| KTT | 1 |  |  |  |  |  |  |  |  | x |  |  |  |  |  |  |  |  |  |  |  |  |  |  |  |  |  |  |  |  |
|  | 2 |  |  | x |  |  |  |  |  |  |  |  |  |  |  |  |  |  |  |  |  |  |  |  |  |  |  |  |  |  |

Supplementary Table S2. Crosses made among plants from self-incompatible populations of *Arabidopsis lyrata*. Population codes follow those from Foxe et al. (2010). Numbers refer to specific plants sourced from each of those populations.

|  |  |  |  |  |  |  |  |  |  |  |  |  |  |  |  |  |  |  |  |  |  |  |  |  |  |  |  |
| --- | --- | --- | --- | --- | --- | --- | --- | --- | --- | --- | --- | --- | --- | --- | --- | --- | --- | --- | --- | --- | --- | --- | --- | --- | --- | --- | --- |
|  | FATHER | IND | | | MAN | | | PCR | | | | PIN | | | | | SBD | | | | | | | | TSS | | |
| MOTHER |  | 10 | 15 | 20 | 8 | 12 | 14 | 5 | 12 | 17 | 20 | 3 | 8 | 13 | 18 | 23 | 3 | 11 | 18 | 20 | 22 | 24 | 28 | 29 | 12 | 25 | 28 |
| IND | 18 |  |  | x |  |  |  |  |  |  |  |  |  |  |  |  |  |  |  |  |  |  |  |  |  |  |  |
| MAN | 8 |  |  |  |  | x |  |  |  |  |  |  |  |  |  |  |  |  |  |  |  |  |  |  |  |  |  |
|  | 12 | x |  |  |  |  |  |  |  |  |  |  |  |  | x |  |  |  |  |  | x |  |  |  |  |  |  |
|  | 14 |  |  |  |  |  |  |  |  |  | x |  |  |  |  |  |  |  |  |  |  |  |  |  |  |  |  |
|  | 27 |  |  |  |  |  |  |  |  |  |  |  | x |  |  |  |  |  |  |  |  |  |  |  |  |  |  |
| PCR | 1 |  | x |  |  |  |  |  |  |  |  |  | x |  |  |  |  |  |  |  |  |  |  |  |  |  |  |
|  | 5 |  |  |  | X |  |  |  |  |  | x |  |  |  |  |  |  |  |  |  |  |  |  |  |  |  |  |
|  | 11 |  |  |  |  |  |  |  |  |  |  | X |  |  |  |  |  |  |  |  |  |  |  |  |  |  |  |
|  | 14 |  |  |  |  |  |  |  | x |  |  |  |  |  |  |  |  |  |  |  |  |  |  |  |  |  |  |
|  | 17 |  |  |  |  |  |  | x |  |  | x |  |  |  | x |  |  |  |  |  | x |  |  |  |  | x |  |
|  | 20 |  |  |  |  |  | x |  |  | x |  |  |  |  |  |  |  |  |  |  |  |  |  |  |  |  |  |
| PIN | 3 |  |  |  |  |  |  |  |  |  |  |  |  |  |  |  |  |  |  | x |  |  |  |  |  |  |  |
|  | 8 |  |  |  |  |  |  |  |  |  |  |  |  |  |  |  |  | x |  |  |  |  |  |  | x |  |  |
|  | 13 |  |  |  |  |  |  |  |  |  |  |  |  |  | x |  |  |  |  |  |  |  |  |  |  |  |  |
|  | 18 |  |  |  |  |  |  |  |  |  |  |  |  |  |  |  |  |  |  |  | x |  |  |  |  |  |  |
|  | 23 |  |  |  |  |  |  |  |  |  |  |  |  |  | x |  |  |  |  |  |  |  |  |  |  |  |  |
| SBD | 3 |  |  |  |  |  |  |  |  |  |  |  |  |  |  |  |  |  |  |  |  | x |  |  |  |  |  |
|  | 11 |  | x |  |  |  |  |  |  |  |  |  | x |  |  |  |  |  |  |  |  |  |  |  |  |  |  |
|  | 18 |  |  |  | X |  |  |  |  |  |  |  |  | x |  |  |  |  |  |  |  |  | x |  |  |  | x |
|  | 22 |  |  |  |  | x |  |  |  |  |  |  |  |  |  |  |  |  |  |  |  |  |  |  |  |  |  |
|  | 24 |  |  |  |  |  |  |  |  |  |  |  |  |  |  |  | x |  |  |  |  |  |  |  |  |  |  |
|  | 28 |  |  |  |  |  |  |  |  |  |  |  |  |  |  | x |  |  | x |  | x |  |  |  |  |  |  |
| TSS | 6 |  |  |  |  |  |  |  |  |  |  | X |  |  |  |  |  |  |  |  |  |  |  |  |  |  |  |
|  | 17 |  |  |  |  |  |  |  |  |  |  |  |  |  |  |  |  |  |  |  |  |  |  | x |  |  |  |

**Statistical analysis of variation in outcrossing rates among self-compatible and self-incompatible cross types of *Arabidopsis lyrata***

To evaluate differences in outcrossing rates among plants from SC and SI cross types, we used linear mixed-effects model with *t_m_* as the dependent variable, *cross type* (SI × SI_within_, SI × SI_between_, SC × SC_within_, SC × SC_between_) as the independent variable, and *maternal ID* (seed family) as a random grouping variable. We used a contrast-matrix approach to evaluate the overall difference in outcrossing rates between plants from SC versus SI populations using the “contrasts” option in the *lmer* function. Orthogonal contrasts included in the contrast matrix also included a test for differences in outcrossing rates between the SC × SC_within_ and SC × SC_between_ cross types and between the SI × SI_within_ and SI × SI_between_ cross types. Mixed-model parameters were calculated using the *lmer* function from the *lme4* package (Bates et al., 2015) in R and significance of fixed effects (the SC versus SI contrast) was assessed using the *lmerTest* package (v. 3.1-3; Kuznetsova et al. 2017).

Plants from the SC × SC_within_ and the SC × SC_between_ cross types had outcrossing rates of *t*_m_ = 0.53 ± 1.01 SD and 0.59 ± 1.25 SD, respectively. Plants from the SI × SI_within_ and the SI × SI_between_ cross types had outcrossing rates of *t*_m_ = 0.73 ± 1.16 SD and 0.81 ± 0.88 SD, respectively. This variation among cross types was associated with a significant difference in average outcrossing rates between plants from SC versus SI cross types (linear mixed-model parameter estimate for the SC versus SI cross-type contrast: -0.23 ± 0.09 SE, *t* = -2.47, *P* = 0.02). Not surprisingly, contrasts of outcrossing rates between cross types within breeding systems were not associated with significant parameter estimates (SC × SC_within_ versus SC × SC_between_: parameter estimate = -0.04 ± 0.13 SE, *t* = -0.32, *P* = 0.75; SI × SI_within_ versus SI × SI_between_: parameter estimate = -0.08 ± 0.13 SE, *t* = -0.62, *P* = 0.54).

Reference:

Kuznetsova A, Brockhoff PB, Christensen RHB (2017). lmerTest Package: Tests in Linear Mixed Effects Models. Journal of Statistical Software 82: 1-26. doi: 10.18637/jss.v082.i13 (URL:https://doi.org/10.18637/jss.v082.i13).

**Calculation of *π* – the daily probability of geitonogamy per plant**

After each initial visit to a plant with *n* flowers, a pollinator either geitonogamously visits another flower on the same plant with probability *p*_g_, or xenogamously visits a flower on another plant with probability *p*_x_ = 1 – *p*_g_. We assume that once a pollinator visits a particular flower it does not revisit the flower. The pollinator then visits two flowers on the same plant with probability *p*_g_ × *p*_x_, three flowers with probability *p*_g_^2^ × *p*_x_, and so on, visiting all flowers per plant with probability *p*_g_^(^*^n^*^-1)^. The sum of these terms gives the total probability of any geitonogamy per plant per initial pollinator visit and is equal to *p_g_*. For example, as diagrammed below, for a plant with five flowers, the probability of geitonogamy is *π* = *p*_g_ × *p*_x_ + *p*_g_^2^ × *p*_x_ + *p*_g_^3^ × *p*_x_ + *p*_g_^4^ = 1 - *p*_x_ = *p_g_*. This calculation, therefore, reflects the outcome of the first move by a floral visitor after their initial visit, which determines whether there is any geitonogamy (*p_g_*) or not (*p_x_*) - later moves by floral visitors are irrelevant for this (although they may affect the intensity of geitonogamy, which we have not considered).

Like any model, our estimates of the rate of geitonogamy are a simplification and a key assumption was that each pollinator movement was independent of the number of flowers on an inflorescence. It is impossible to evaluate whether this assumption was realistic for our study without more detailed observations. Another key assumption was that the probability of geitonogamous pollen transfer did not increase with the number of within-inflorescence pollinator moves (and thus did not depend on visit duration). Future work should test these assumptions in more detail and inform any expansions or extensions of the model.

= 1 - *p*_x_ *= p*_g_


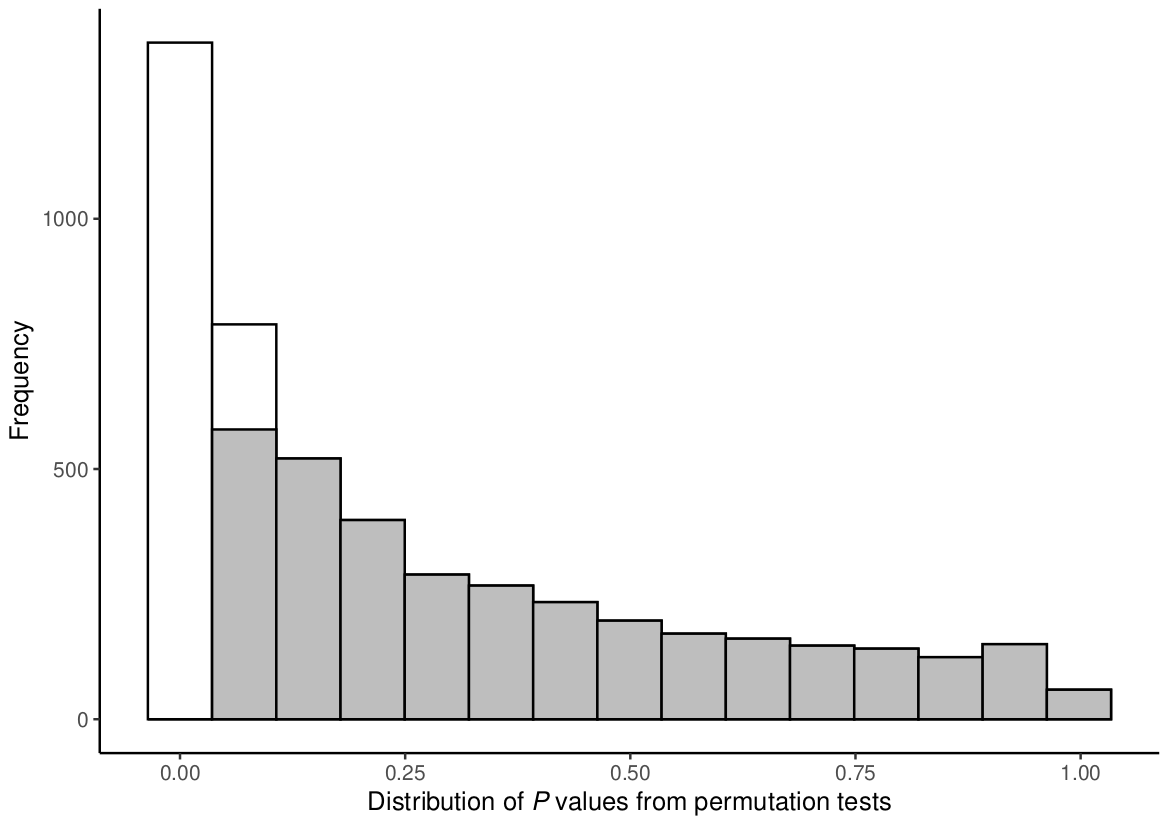


Figure S1. Distribution of *P*-values from permuted, linear mixed-model (LMM) tests of the difference in average outcrossing rates (*t*_m_) between plants sourced from SC versus SI maternal lineages of plants of *Arabidopsis lyrata* grown in a common garden. Permutation tests were conducted by randomly resampling (with replacement) outcrossing rate estimates from 800 individual seeds (400 seeds from SC plants, 400 seeds from SI plants) and using those resampled data, calculated 5000 LMMs and recorded the *P*-values associated with the *breeding system* fixed effect (see Methods for additional details). The grey portion of the distribution indicates the subset of permuted LMMs for which the difference in outcrossing rates was not statistically significant.
